# 

**Published:** 1864-01

**Authors:** 


### DENTAL REGISTER

### OF THE WEST.

EDITED BY

J. TAFT AND GEO. WATT.

JANUARY.—1864.

**MONTHLY:**
**Three Dollars per Annum, in advance.**

CINCINNATI:
J. TAFT, PUBLISHER.

J. TAFT, DENTIST, 172 RACE STREET.

